# Diagnostic challenges in an aggressive case of peripheralizing marginal zone lymphoma

**DOI:** 10.1002/ccr3.3395

**Published:** 2020-10-18

**Authors:** Sujal I. Shah, Christopher R. D’Angelo, Jeremy D. Kratz, David T. Yang

**Affiliations:** ^1^ Department of Pathology University of Wisconsin School of Medicine and Public Health Madison WI USA; ^2^ Department of Medicine Section of Hematology/Oncology University of Wisconsin School of Medicine and Public Health Madison WI USA

**Keywords:** B‐cell lymphoma, cytogenetics, mate‐pair sequencing, splenic marginal zone lymphoma, t(2;7)

## Abstract

B‐cell lymphomas with atypical presentation or immunophenotype pose diagnostic challenges. Conventional ancillary tests (cytogenetics, FISH) can help, but have technical limitations. New technologies such as mate‐pair sequencing (MPSeq) offer a route around these technical limitations.

## Graphical abstract

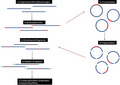

## 1 INTRODUCTION

Evaluation of B‐cell lymphomas benefits from cytogenetic analysis, however, limitations in conventional karyotyping and fluorescence in situ hybridization curtail their clinical utility. This case highlights value of cytogenetics in diagnosis of atypical B‐cell lymphoma and discusses the benefits of new technology that can overcome the limitations of conventional cytogenetic studies.

Mature B‐cell neoplasms are relatively common neoplasms that can occasionally pose diagnostic challenges. In these cases, cytogenetics can be a useful tool for both diagnosis and prognosis. Common ancillary cytogenetics studies—conventional karyotyping and fluorescence in situ hybridization (FISH)—are almost universally available; however, both modalities have limitations that can curtail their utility in evaluation of mature B‐cell neoplasms with atypical presentation or phenotypes. We present a case of peripheralizing marginal zone lymphoma with an atypical immunophenotype and clinical presentation and discuss the advantages and limitations of both conventional karyotyping and FISH in this setting. We also discuss how new next‐generation sequencing (NGS) based methods, specifically referencing mate‐pair sequencing, can bridge the gap between these limitations and why they should be considered in the toolkit of ancillary cytogenetic tests.

## 2 CASE PRESENTATION

A 68‐year‐old Caucasian man with a past medical history of obstructive sleep apnea, hypertension, and gout presented to a community hospital with shortness of breath, night sweats, and fatigue. His dyspnea had significantly worsened, in addition to experiencing a 38 lb. weight loss over the prior 3 months. Physical examination revealed splenomegaly and lower extremity edema without lymphadenopathy. Blood work revealed a markedly elevated WBC count of 423 K/uL, along with hyperkalemia (7.2 mmol/L), hypercalcemia (11.8 mg/dL), elevated liver enzymes (ALT 55 U/L, AST 91 U/L, alkaline phosphatase 236 U/L), lactic acidosis (5.0 mmol/L), and elevated creatinine (1.47 mg/dL). He was transferred for further workup and care.

**Figure 1:**
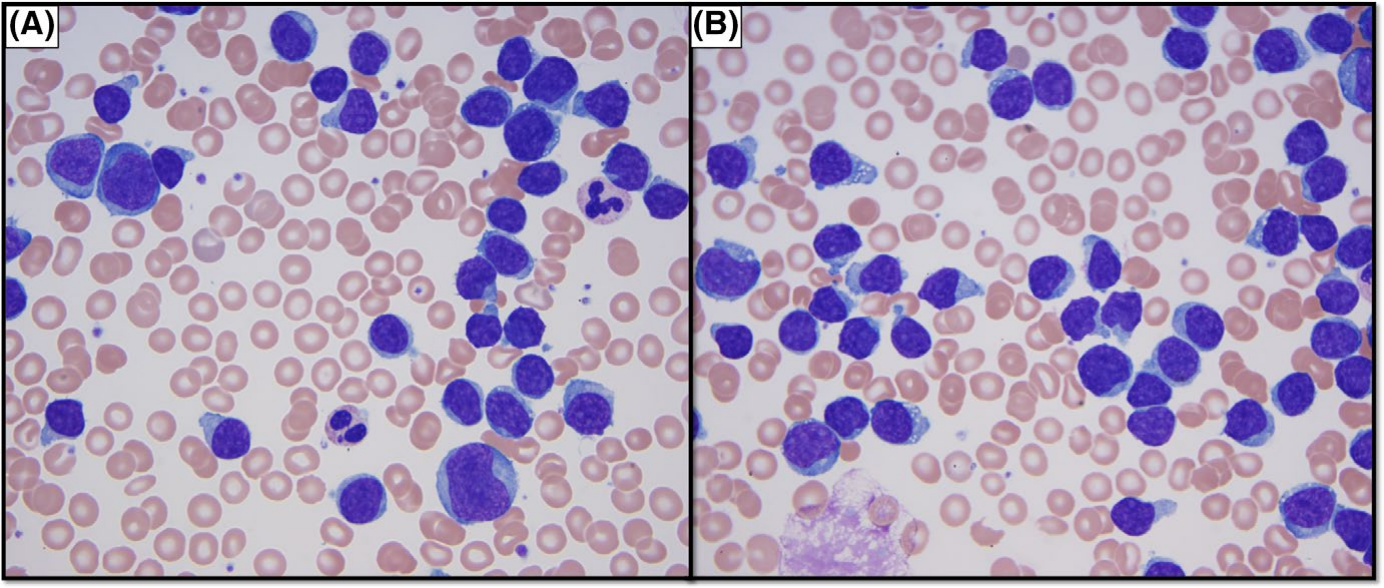
Peripheral blood smear. Abundant cells with high nucleus/cytoplasm ratio and variably prominent nucleoli

The initial presenting symptoms, laboratories, and physical examination findings in this patient raised concern for a hematologic malignancy with spontaneous tumor lysis syndrome. Given the markedly elevated peripheral WBC count, the initial differential diagnosis list included acute leukemia, chronic leukemia, and peripheralizing lymphoma. (Causes of neoplastic leukocytosis are listed in Table 1.) Initial examination of the peripheral smear was concerning for atypical lymphocytes versus possible blasts, with cells showing large round nuclei and scant cytoplasm (Figure 1). Bilobed nuclei or Auer rods were not appreciated.

**Table 1:** Common Neoplastic Causes of Leukocytosis

Common Neoplastic Causes Leukocytosis	Predominant Cell Type
Chronic lymphocytic leukemia	Mature lymphocytes
Chronic myeloid leukemia	Bands and segmented neutrophils
Acute lymphoblastic lymphoma	Lymphoid blasts
Acute myeloid leukemia	Myeloid blasts (& blast‐equivalents)

The patient had no known history of underlying kidney disease, with baseline creatinine of 1.0 mg/dL. Given his elevated creatinine at presentation, along with the additional metabolic derangements of hyperkalemia, hypercalcemia, and lactic acidosis, the initial working diagnosis was tumor lysis syndrome with acute kidney injury.

Hematopathology review of the peripheral smear revealed a predominance of intermediate‐sized lymphoid cells with scant cytoplasm, smooth nuclear contours, and condensed nuclear chromatin comprising 92% of the nucleated cells. No Auer rods or cytoplasmic granules were seen.

With these findings, chronic leukemia or peripheralizing lymphoma were considered the most likely underlying malignancy, with acute leukemia considered less likely based on morphologic assessment of the leukocytosis. (Common neoplastic causes of marked lymphocytosis are listed in Table 2.)

**Table 2:** Common Neoplastic Causes of Marked Lymphocytosis

Neoplastic Causes of Marked Lymphocytosis (WBC > 100 K/μL)
Chronic lymphocytic leukemia
B‐cell prolymphocytic leukemia
T‐cell prolymphocytic leukemia
Peripheralizing lymphoma

The morphology of the circulating atypical lymphocytes was not classic for chronic lymphocytic leukemia (CLL) or B‐cell prolymphocytic leukemia, heightening the concern for T‐cell prolymphocytic leukemia or a peripheralizing lymphoma.

### 2.1 Investigations

**Figure 2:**
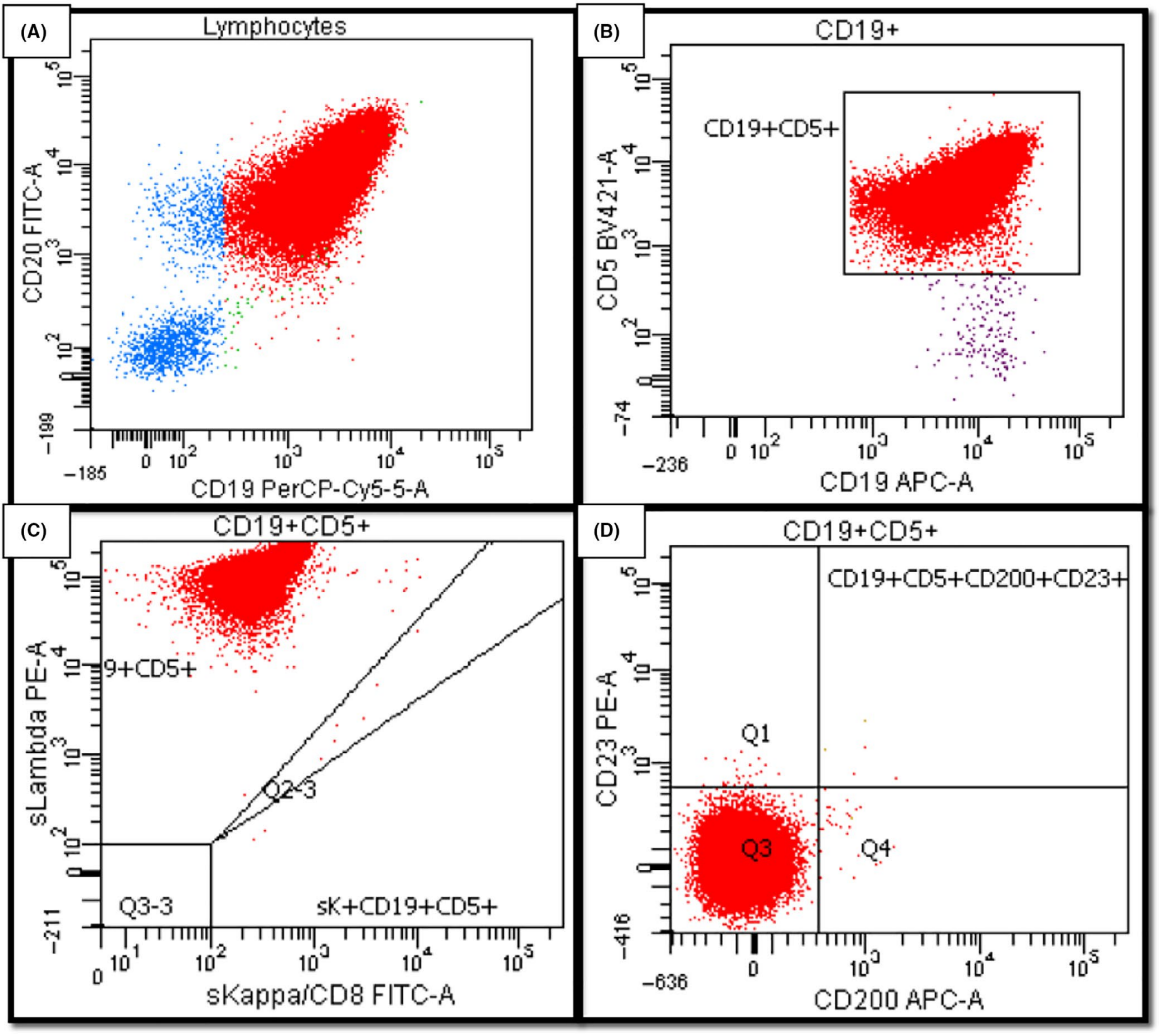
Peripheral blood flow cytometry. A, The atypical cells (in red) are positive by both CD20 and CD19. B, The cells are CD5‐positive. C, The cells are monoclonal, showing strong expression for lambda light chain. D, The cells are negative by both CD200 and CD23

Flow cytometry studies revealed the circulating lymphocytes were clonal B cells that expressed CD45, bright CD20, CD19, CD5, and bright lambda light chains without CD10, CD23, CD200, or CD38 (Figure 2). The flow was interpreted as a CD5‐positive B‐cell lymphoproliferative disorder, favoring mantle cell lymphoma, as the immunophenotype was not characteristic for CLL.

Of the CD5‐positive B‐cell leukemias, CLL and peripheralizing mantle cell lymphoma are the most common, though occasionally marginal zone lymphomas and follicular lymphomas also can express CD5^1^, ^2^ (Table 3). The additional findings on flow cytometry in this case (CD23‐negative, CD200‐negative, bright immunoglobulin, and bright CD20 expression) are not characteristic of CLL, bringing mantle cell lymphoma to the top of the differential list, with an atypical CLL less likely. Fluorescence in situ hybridization (FISH) studies for t(11;14) *CCND1/IGH* fusion were ordered for confirmation, with suggestion to also perform a CLL FISH panel should *CCND1/IGH* fusion be negative.

**Table 3:** B‐cell Lymphomas with CD5 expression

B‐cell lymphomas with CD5‐expression	% of cases^24^
CLL/SLL	80%‐100%
Mantle cell lymphoma	95%
Lymphoplasmacytic lymphoma	10%‐40%
Splenic marginal zone lymphoma	20%
Nodal marginal zone lymphoma	10%
De novo DLBCL	10%

**Figure 3:**
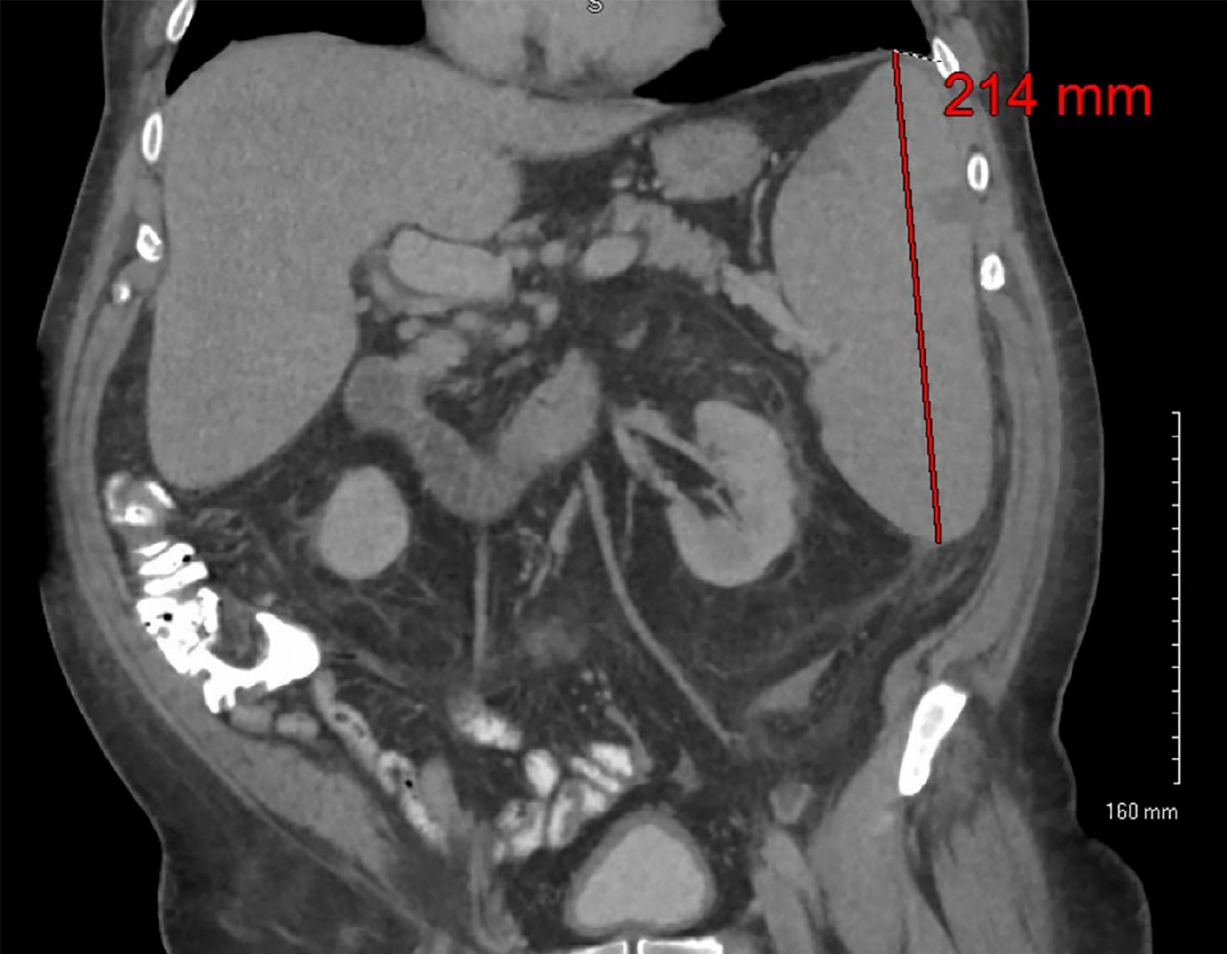
CT Abdomen/Pelvis from diagnosis, revealing splenomegaly without appreciable additional mesenteric adenopathy

Peripheralizing mantle cell lymphoma is frequently a result of an aggressive leukemic transformation of the underlying disease, which in this case fits with the clinical presentation of tumor lysis syndrome. However, leukemic transformation on mantle cell lymphoma should be associated with diffuse lymphadenopathy. In this case, staging CT scans of the chest, abdomen, and pelvis confirmed the physical examination findings of splenomegaly, measuring up to 21 cm in the intracranial coronal dimension, with peripheral wedge‐shaped infarcts, but no frank adenopathy was noted (Figure 3). Alternatively, an indolent subtype of mantle cell lymphoma has been identified that typically presents with isolated circulating disease without lymphadenopathy, but as the name suggests, patients tend to be asymptomatic and have minimal lymphocytosis.^3^, ^4^

To evaluate further, a bone marrow aspirate and core biopsy were performed. The bone marrow aspirate showed increased numbers of small mature lymphocytes without an increase in plasma cells or plasmacytoid lymphocytes. The biopsy showed increased numbers of small lymphocytes both in an infiltrative pattern and forming loose lymphoid aggregates, occupying approximately 20% of the marrow space. Immunohistochemical staining showed the lymphocytes expressed CD20 and PAX5. No areas concerning for large cell transformation were identified.

**Figure 4:**
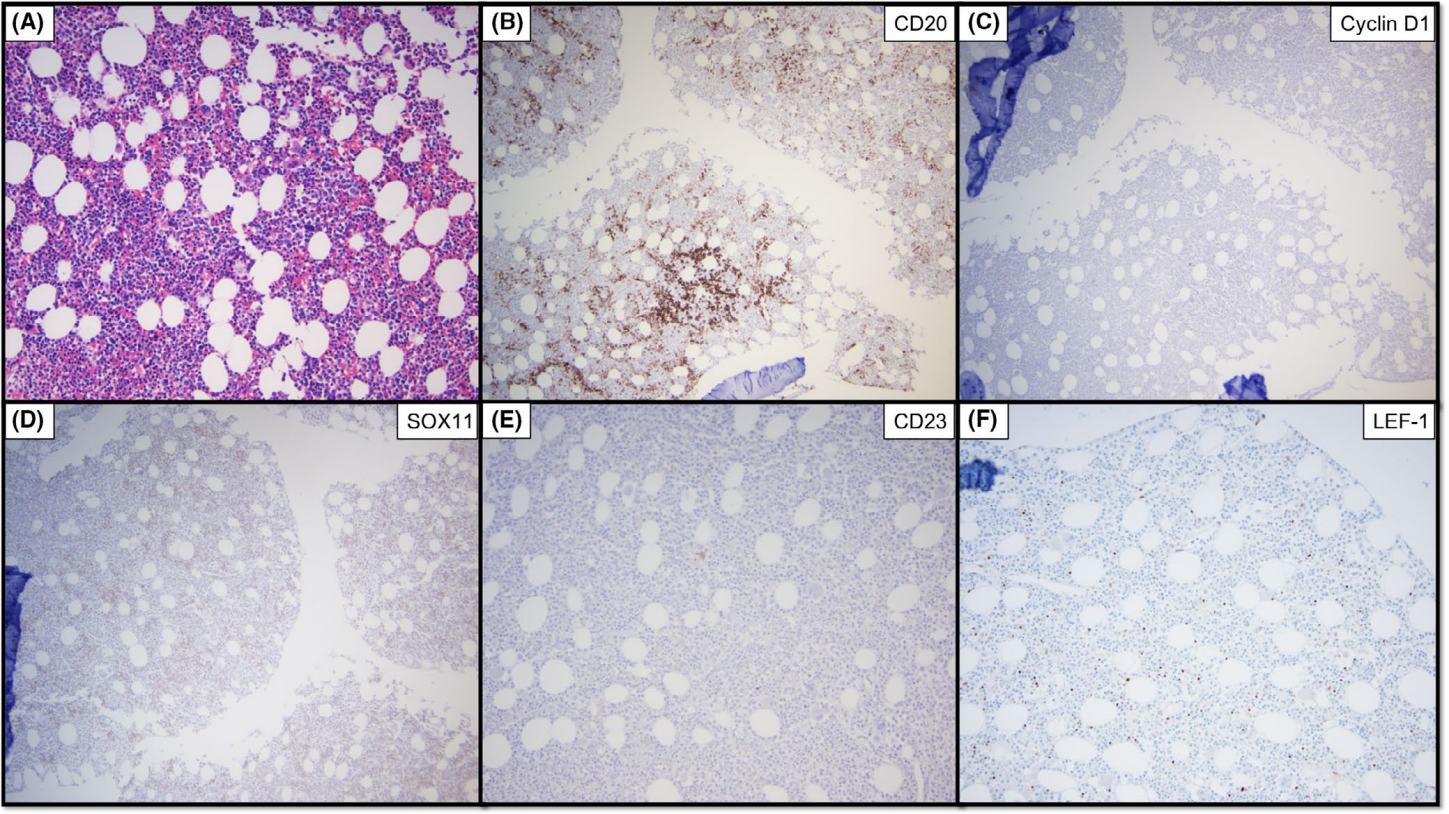
Splenic marginal zone lymphoma involving bone marrow. A, Hematoxylin and eosin stained section showing infiltrate of small, mature lymphocytes (20x). B, Lymphocytes stain positive for CD20 (10x). C, Lymphocytes are negative for Cyclin D1 (10x). D, Lymphocytes are negative for SOX11 (10x). E, Lymphocytes are negative for CD23 (20x). F, Lymphocytes are negative for LEF‐1 (20x)

Given the suspicion for mantle cell lymphoma, Cyclin D1 stain was performed—and came back negative. A small proportion of mantle cell lymphoma cases are known to be Cyclin D1 negative,^5^, ^6^ therefore SOX11 staining was subsequently performed—and was also negative. Immunostain for p53 was initially ordered given the leading differential of mantle cell lymphoma and was also negative. These results, while not completely excluding mantle cell lymphoma, made the diagnosis highly unlikely. Likewise, CLL also remained unlikely based on the previous flow cytometry results, as well as a lack of CD23 or LEF1 expression as evaluated by immunohistochemistry on the core biopsy specimen (Figure 4). While considering this diagnostic dilemma, karyotyping results performed on the bone marrow aspirate became available.

Conventional karyotyping revealed an abnormal clone with highly complex structural and numerical abnormalities, comprising 16 out of 20 metaphase cells. Some of the abnormalities identified included t(2;7) and t(1;12), along with del(9p), del(13q), loss of Y, iso(8q) and iso(17q). Of note, t(11;14) *CCND1/IGH* was not identified and FISH for t(11;14) was also negative, further confirming that the neoplastic process was not mantle cell lymphoma. Review of published literature revealed a few case reports and case series of t(2;7)(p11.2;q22) being associated with marginal zone lymphoma,^7^, ^8^, ^9^, ^10^ including splenic marginal zone lymphoma.^11^, ^12^ While marginal zone lymphoma typically does not express CD5, expression has been identified in up to 25% of cases.^1^, ^13^, ^14^

Not only were the karyotyping results helpful in establishing the diagnosis of marginal zone lymphoma, they also were prognostically significant. Isochromosome 17q results in deletion of one *TP53* allele on the short arm of chromosome 17 and mutation analysis, ordered on peripheral blood when the diagnosis of mantle cell lymphoma was being entertained, revealed that the remaining *TP53* allele showed a likely pathogenic truncating mutation: p.Glu258Thrfs*86. The presence of biallelic *TP53* derangement likely contributed to the complex karyotype and aggressive clinical presentation of this B‐cell lymphoma. Interestingly, truncating *TP53* mutations are associated with lack of p53 immunohistochemical staining,^15^ as demonstrated in this case.

Overall, the constellation of clinical, immunophenotypic, molecular, and cytogenetic findings in this challenging case, resulted in a final diagnosis of CD5‐positive B‐cell lymphoproliferative disorder with t(2;7)(p11.2;q22) and *TP53* mutation, favoring splenic marginal zone lymphoma.

### 2.2 Treatment

The patient received intravenous fluids, allopurinol, and rasburicase for treatment of tumor lysis syndrome with improvements in his electrolyte derangements and creatinine. Based on the working diagnosis of B‐cell lymphoma, he received an empiric course of steroids while awaiting formal diagnosis for definitive treatment. Additionally, based on this working diagnosis, the patient was treated with a single cycle of bendamustine and rituximab chemoimmunotherapy. This resulted in a transient improvement in his peripheral lymphocytosis, but was also complicated by a severe desquamating skin rash diagnosed as Stevens‐Johnson syndrome. He recovered and, based on the poor prognosis associated with *TP53* mutated B‐cell lymphomas,^16^ went on to receive combination venetoclax and obinutuzumab, which resulted in complete resolution of his peripheral lymphocytosis after 1 cycle. He has since continued on treatment with venetoclax and obinutuzumab and is now approximately 3 months out from his initial diagnosis.

## 3 DISCUSSION

This case illustrates the benefit of additional ancillary testing in diagnosing a peripheralizing lymphoma in an acutely ill patient that was not definitively classifiable based on flow cytometry, bone marrow histology, and immunohistochemistry. Peripheral blood revealed a marked lymphocytosis, comprised of small, mature lymphocytes. Flow cytometry revealed an atypical, CD5‐positive population of monoclonal B‐lymphocytes; however, additional markers and subsequent immunohistochemical stains performed on the bone marrow biopsy were not in keeping with a diagnosis of either chronic lymphocytic leukemia or mantle cell lymphoma.

It was fortunate that evaluable neoplastic cells in metaphase could be obtained from a bone marrow aspirate for conventional karyotyping, which revealed a complex karyotype that included a t(2;7) translocation, an anomaly that has been associated with marginal zone lymphoma including a few cases of splenic marginal zone lymphoma. In conjunction with the clinical findings of splenomegaly without significant adenopathy, peripheralizing splenic marginal zone lymphoma was favored as the final diagnosis. In addition, isochromosome (17q) found on karyotyping combined with *TP53* mutation detected by next‐generation sequencing confirmed a biallelic *TP53* derangement, likely contributing to the patient's aggressive presentation. These ancillary studies proved invaluable in reaching a final diagnosis and risk‐stratification in this case.

In mature B‐cell neoplasms, cytogenetic analysis can be a useful tool for both diagnosis and prognosis. This case highlights the advantage of the unbiased nature of conventional karyotyping over FISH, where it was very unlikely that FISH analysis for t(2;7) would have been pursued. However, conventional karyotyping requires isolation of mitotically active, dividing cells, which are difficult to grow in vitro in low‐grade lymphomas and as a result, is typically non‐contributory in lymphoma diagnosis.^17^ In addition, the resolution of conventional karyotyping is approximately 5‐10 Mb, with smaller insertions/deletions/translocations remaining ‘cytogenetically cryptic’ by conventional karyotype. In contrast, FISH does not require presence of living, dividing cells and has a resolution of approximately 100 Kb.^18^ The limitation of FISH is that it is a targeted methodology, where specific probes must be selected to detect specific insertion, deletion, or translocations that are suspected.

**Figure 5:**
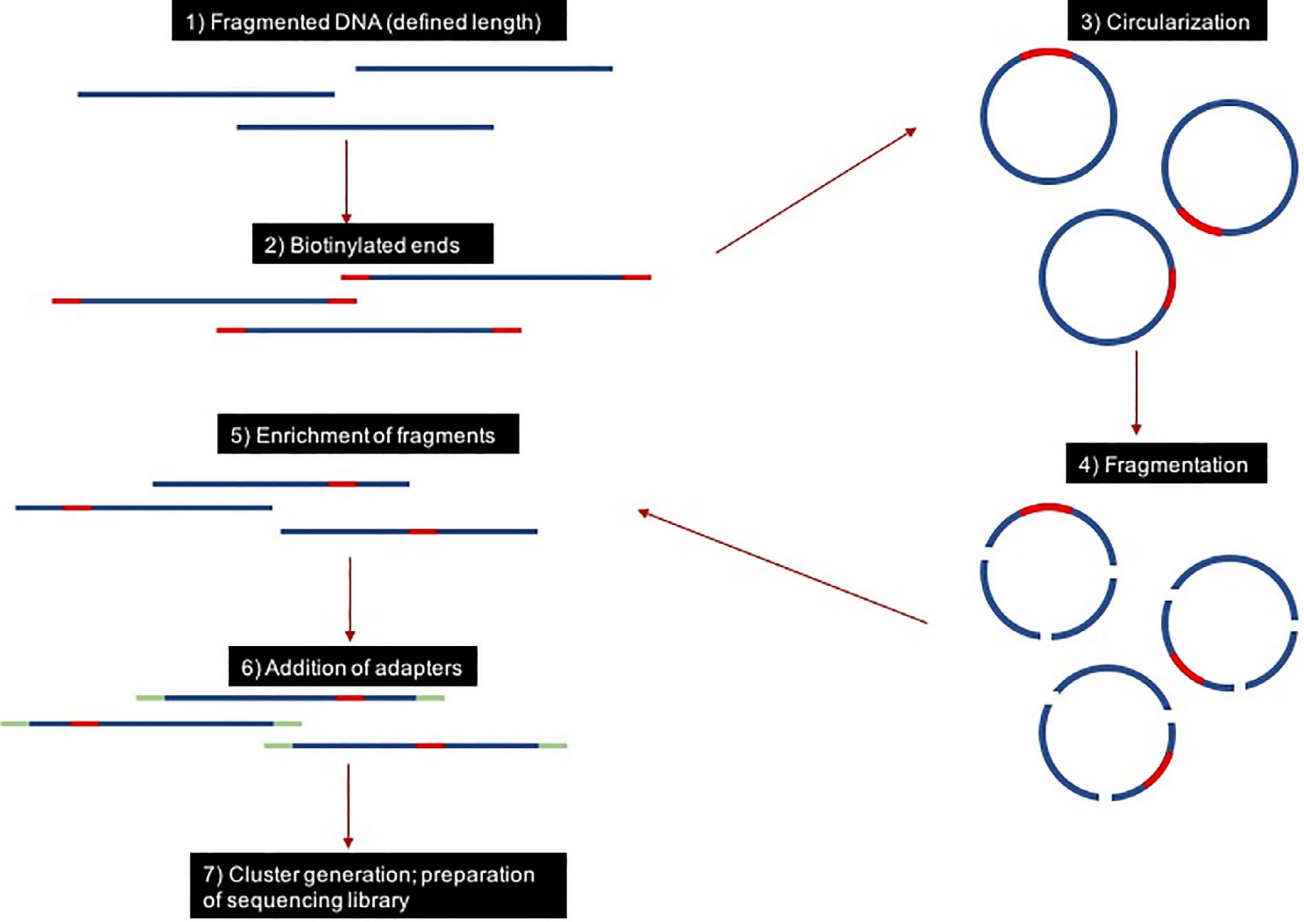
Mate‐pair sequencing. 1) Genomic DNA is fragmented to 2‐5 Kb fragments and 2) the ends of the fragments are biotinylated. 3) Fragments are then circularized to bring the biotinylated ends together and then 4) the circularized DNA is fragmented to 200‐500 bp fragments. 5) Biotinylated fragments are captured and 6) the library preparation is completed by adding tags and adaptor sequences to the fragment ends for identification during multiplexing and hybridizing to the flow cell for subsequent 7) cluster generation. Because the fragment was originally circularized, the sequence obtained should be mapped to two areas of the genome approximately 2‐5 Kb apart. A genomic duplication or deletion will result in an unexpected distance between the sequences on a fragment. A translocation will result in the mapping of two unexpected chromosomes on the same fragment. An inversion will result in mapping an unexpected orientation in a portion of the sequence of a fragment

Newer ancillary studies are now available that can bridge the gap between the limitations of conventional karyotyping and FISH. An example is mate‐pair sequencing (MPseq), which utilizes next‐generation sequencing technology to provide unbiased, relatively high‐resolution DNA analysis for insertions, deletions, and translocations. Mate‐pair sequencing involves the formation of libraries, which are produced by initially generating kilobase‐sized fragments. Markers are added to the ends of these fragments, and the fragments are circularized and ligated. Non‐circularized DNA molecules are removed with exonucleases, and the residual, circularized molecules are sheared into small, approximately 500bp fragments (Figure 5). The library of fragments, purified using magnetic beads, is assessed by paired‐end next‐generation sequencing. The resulting 100‐150 base pairs of sequence generated from these two end fragments can then be aligned to a reference genome. The mate‐pairs provide information about the entire region between the two fragments, known as bridged coverage.^19^ As a result, MPseq can be used to detect rearrangements genome‐wide, both balanced and unbalanced structural and copy‐number abnormalities,^20^ with significantly greater resolution and sensitivity than conventional karyotype without the need for mitotically active cells.^21^ Additionally, MPseq allows for comprehensive evaluation using a single assay, as opposed to large panels of independent FISH probes.^22^

## 4 CONCLUSION

Cytogenetic studies are a useful tool in instances where mature B‐cell neoplasms prove to be diagnostically challenging, with the results providing both diagnostic and prognostic benefit. To this end, conventional karyotyping and fluorescence in situ hybridization (FISH) are almost universally available; however, both methodologies have limitations that can impede on their utility in evaluation of low‐grade and mature B‐cell neoplasms. Newer testing methodologies, such as MPseq, provide opportunities to work around these limitations.

While we were fortunate that conventional karyotyping was successful in this case, it helps to highlight the benefits of employing these new technologies to aid not only in the diagnosis of challenging cases, but also possibly in more comprehensively characterizing the chromosomal anomalies of otherwise seemingly routine cases.^22^, ^23^

## CONFLICT OF INTEREST

None declared.

## AUTHOR CONTRIBUTIONS

SS performed the literature search and drafted the initial manuscript. CD provided the treatment section for the manuscript. SS and JK provided pathologic and clinical images, respectively. DY and SS were involved in the pathologic diagnosis of the case. CD and JK were involved in the clinical care of the patient. CD and DY critically revised the manuscript. All authors read and approved the final manuscript and agree to be fully accountable for ensuring the integrity and accuracy of the work.

## ETHICAL STANDARDS

Informed consent was obtained from the patient discussed in this report. Our institution does not require ethical approval for case reports.
